# Intranasal hydrogels for Alzheimer’s disease: from nasal-interface engineering to brain targeting and translational readiness

**DOI:** 10.3389/fphar.2026.1960603

**Published:** 2026-09-29

**Authors:** Hossein Omidian, Luigi X. Cubeddu

**Affiliations:** Department of Pharmaceutical Sciences, Barry and Judy Silverman College of Pharmacy, Nova Southeastern University, Fort Lauderdale, FL, United States

**Keywords:** AD, brain pharmacokinetics, intranasal hydrogel, nose-to-brain delivery, translational pharmaceutics

## Abstract

Intranasal hydrogels offer a strategy to address rapid mucociliary clearance, limited nasal residence, difficult payload properties, and insufficient brain exposure in Alzheimer’s disease (AD). This Review evaluates hydrogel and hydrogel-associated systems across formulation architecture, nasal-interface performance, brain pharmacokinetics, pharmacodynamics, safety, and translation. Selected systems improved deposition or retention, controlled release, permeation, and central exposure relative to relevant comparators, with some also showing convergent behavioral and disease-relevant biological effects in preclinical models. The strongest evidence comes from matched comparisons that distinguish route effects from the incremental contributions of carriers and hydrogels. However, rheology, deposition, targeting metrics, statistical reporting, complete-product stability, repeated-dose safety, device performance, and human reproducibility remain inconsistently characterized. Overall, the evidence supports intranasal hydrogels as a credible preclinical platform for brain-directed AD drug delivery, but not as clinically validated therapy, and favors standardized, device-integrated, comparator-resolved development.

## Graphical Abstract

**Figure:**
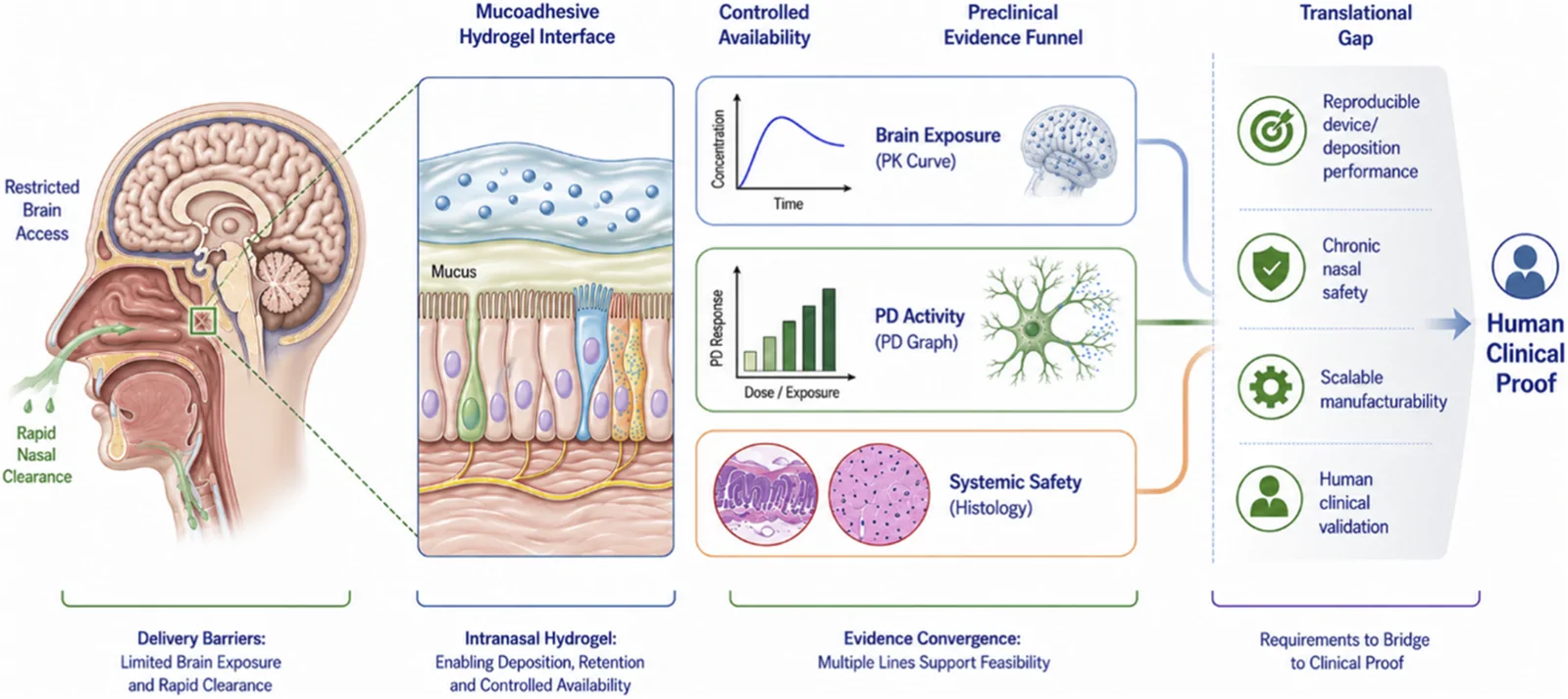

## 1 Introduction

AD drug delivery is constrained not only by limitations of pharmacotherapy (Agraharam et al., 2022) but also by the difficulty of achieving sufficient, sustained, and tolerable brain exposure. Candidate and established agents may be limited by low oral bioavailability, first-pass metabolism, short half-life, physicochemical instability, poor aqueous solubility, peripheral adverse effects, or restricted brain penetration (Achmad et al., 2024; Ansari et al., 2024; Aslam et al., 2026; Choudhary et al., 2024; Hinge et al., 2023; Ribeiro et al., 2022; Shoman et al., 2026; Varshney et al., 2026; Zoupanou et al., 2025). Intranasal administration offers a non-invasive route with potential access to the central nervous system through olfactory- and trigeminal-associated pathways, but route selection alone does not fully address rapid mucociliary clearance, inadequate mucosal residence, formulation-dependent regional deposition, or challenges related to payload stability and release (Abou-Taleb et al., 2024; Adnet et al., 2020; Cunha et al., 2022; Di Lelio et al., 2026; Huang et al., 2024; Nagaraja et al., 2021; Puranik et al., 2026; Ribeiro et al., 2022; Wavikar et al., 2017; Wavikar and Vavia, 2015).

Hydrogels are therefore best considered delivery-enabling dosage forms rather than therapeutic interventions in themselves. Their value lies in controlling administration behavior, increasing mucosal contact, resisting premature washout, prolonging local residence, modulating release, and localizing active agents or drug-loaded carriers at the nasal interface (Abou-Taleb et al., 2024; Cunha et al., 2022; Huang et al., 2024; Nagaraja et al., 2021; Wavikar et al., 2017; Wavikar and Vavia, 2015). For clinically established symptomatic agents such as rivastigmine and donepezil, the central development question is whether a hydrogel-associated nasal formulation improves delivery relative to oral, systemic, or simple intranasal comparators (Abouhussein et al., 2018; Achmad et al., 2024; Al Harthi et al., 2019; Gu et al., 2020; Mathure et al., 2025; Wavikar et al., 2017). For experimental small molecules, biologics, enzymes, extracellular vesicles, and multifunctional materials, the hydrogel may enable the delivery of payloads otherwise limited by solubility, stability, retention, or delivery complexity (Di Lelio et al., 2026; Huang et al., 2024; Humpel, 2021; Liu et al., 2024; Özyilmaz, 2026; Ribeiro et al., 2022).

This Review evaluates the field across a development continuum spanning therapeutic rationale, formulation architecture, nasal-interface performance, brain pharmacokinetics, pharmacodynamics, safety, and human translation. Rather than ranking polymers or carrier classes, the synthesis emphasizes two questions: whether the hydrogel corrects a defined delivery failure and whether the evidence distinguishes improved delivery from therapeutic efficacy. Particular attention is given to the coupled trade-offs among sprayability, gelation, viscosity, retention, release, permeation, and deposition, as well as to the strength of comparator-based brain-targeting claims. The central conclusion is deliberately bounded: selected systems report preclinical delivery and biological activity, but the evidence summarized here does not establish clinical efficacy.

## 2 Methods and scope of evidence synthesis

### 2.1 Evidence source, search record, and study selection

The literature search was conducted in Web of Science, with the final search performed around 4 August 2026. A focused, high-precision Topic search was used to identify original research addressing Alzheimer’s disease (AD), intranasal or nose-to-brain delivery, hydrogel or hydrogel-associated dosage forms, and experimentally evaluated formulation, transport, or pharmacokinetic outcomes *((“Alzheimer’s disease” OR “Alzheimer disease” OR Alzheimer) AND (intranasal OR “nose-to-brain” OR “nose to brain”) AND (hydrogel* OR “*in situ* gel*” OR “*in-situ* gel*” OR thermosensitive OR thermoresponsive OR mucoadhesive OR nanoemulgel*) AND (pharmacokinetic* OR biodistribution OR “brain targeting” OR “brain delivery” OR “brain concentration” OR AUC OR Cmax OR Tmax OR DTE OR DTP OR permeation OR “drug release” OR release OR rheolog* OR viscosity OR mucoadhesion OR deposition OR “*in vivo*” OR “*ex vivo*”)) NOT (retract* OR withdraw* OR editorial* OR “letter to the editor” OR commentary OR “conference abstract” OR “meeting abstract” OR “study protocol”)**.

Original research articles constituted the primary evidence base. Review articles, retracted or withdrawn publications, editorials, letters, commentaries, conference-only records, study protocols, and other non-primary publications were excluded from the primary evidence synthesis. The review was conducted as a focused critical evidence synthesis rather than a prospectively registered systematic review.

The search placed its principal publication emphasis on studies published during 2024–2026 to capture recent advances in formulation design, nose-to-brain delivery, and translational development. No absolute lower publication-year restriction was applied, however, because selected earlier studies were required to establish the development and mechanistic basis of the field. Earlier publications were retained only when they provided directly relevant evidence concerning hydrogel formulation design; gelation, rheology, mucoadhesion, retention, release, permeation, or regional deposition; pharmacokinetics, biodistribution, or brain-targeting metrics; matched comparator evidence capable of distinguishing route-, carrier-, or hydrogel-associated effects; or mechanistic evidence necessary to interpret intranasal transport or the development of hydrogel-based nose-to-brain delivery.

Studies were considered core evidence when they directly addressed intranasal hydrogel-mediated or hydrogel-associated delivery in AD or a directly relevant neurodegenerative context. Particular emphasis was placed on studies in which the hydrogel constituted the final intranasal dosage form or served as a functionally integral depot for a separately engineered carrier, and in which formulation performance, brain delivery, pharmacokinetics, or AD-relevant pharmacodynamic outcomes could be evaluated. Evidence from non-AD psychiatric, perioperative, chemotherapy-related, sepsis-associated, or other cognitive-disorder models was not used to support AD-specific therapeutic conclusions. Selected studies from these contexts were retained only when they informed general principles of nasal formulation, retention, transport, deposition, or brain targeting (Elhelw et al., 2026; Fei et al., 2025; Li et al., 2024; Parmar et al., 2026; Xie Z. et al., 2026; Zhuang et al., 2024).

The same distinction was applied to route-mechanistic studies and adjacent delivery technologies. Intranasal systems that did not satisfy the operational definition of a final hydrogel or hydrogel-associated dosage form were not treated as core evidence for hydrogel-specific effects. These included polymeric-gel or polymer-derived transport studies involving melatonin or gellan-derived oligosaccharides (Babu et al., 2011; Makani et al., 2016; Murphy et al., 2018), as well as thermoresponsive or mucoadhesive nanoparticulate insulin systems without a separate final hydrogel depot (Dwivedi et al., 2013; Khan et al., 2026). Standalone nasal nanoparticles, microparticles, powders, or microemulsions lacking an integral hydrogel matrix, together with transdermal or facial-skin microneedle systems, were therefore classified as adjacent technologies rather than primary intranasal hydrogel evidence (Bhavna et al., 2014; Di Lelio et al., 2026; Elmorsy et al., 2026; Espinoza et al., 2018; Guimaraes et al., 2022; Jadhav and Mishra, 2022; Kouhjani et al., 2025; Meng et al., 2018; Permana et al., 2024; Ruan et al., 2024; Saini et al., 2021; Saladini et al., 2013).

For this Review, a “hydrogel-associated system” is operationally defined as an intranasal formulation in which a gel-forming, mucoadhesive, thermoresponsive, ion-responsive, pH-responsive, chemically crosslinked, or biologically responsive matrix is integral to the administration, localization, retention, release, or performance of the active payload or its carrier. This definition includes both direct drug-loaded gels and carrier-in-gel systems in which nanoparticles, vesicles, lipid carriers, nanocrystals, biologics, enzymes, or extracellular vesicles are incorporated into a final hydrogel depot. It excludes carrier systems in which a hydrogel is absent or peripheral to the final intranasal dosage form.

### 2.2 Synthesis framework and terminology

The evidence was synthesized comparatively rather than through sequential study descriptions. Formulation findings were grouped by architecture and by the function of the hydrogel at the nasal interface; pharmacokinetic evidence was interpreted according to endpoint directness and comparator quality; and therapeutic evidence was assessed according to convergence among behavioral, molecular, biochemical, and histopathological outcomes.

Pharmaceutical-development terminology is used hierarchically. The quality target product profile (QTPP) refers to the prospective product objectives required for a viable intranasal brain-directed dosage form. Critical material attributes (CMAs) are properties of drug substances, polymers, lipids, surfactants, carriers, and excipients that can influence product performance. Critical process parameters (CPPs) are manufacturing variables, such as mixing, homogenization, sonication, or other processing conditions, whose variation can affect the resulting product. Critical quality attributes (CQAs) are measurable product properties that require control to assure appropriate quality. By contrast, sprayability, gelation behavior, rheology, mucoadhesion, retention, release, permeation, and deposition are treated here primarily as formulation-performance attributes unless a specific development program has formally designated them as CQAs. Brain maximum concentration (C_max_), time to maximum concentration (T_max_), area under the concentration–time curve (AUC), bioavailability, biodistribution, and targeting indices are pharmacokinetic or distribution measures, whereas cognitive outcomes, biomarker changes, histopathology, repeated-dose tolerability, device reproducibility, manufacturability, and usability are pharmacodynamic, safety, or translational endpoints. This separation prevents efficacy from being used as a substitute for formulation quality.

Inferential statistics were not reported consistently in reviewed studies. Some studies provide p values, standard deviations, or sample-size information, whereas others report point estimates or qualitative significance without full inferential details (Camlik et al., 2024; Cunha et al., 2022; Hard et al., 2025; Huang et al., 2024; Xie R. Y. et al., 2026). The synthesis therefore emphasizes effect direction, comparator structure, and convergence rather than treating the absence of a reported p value in the master table as evidence of either significance or non-significance.

## 3 Therapeutic rationale: match the hydrogel to the delivery limitation

A coherent hydrogel-development program begins with a specific delivery problem rather than a preferred material. Across the evidence corpus, recurring barriers include inadequate brain exposure, first-pass or hepatic loss, poor solubility or instability, rapid mucociliary clearance, insufficient nasal residence, systemic adverse effects, and practical limitations of conventional dosing (Abou-Taleb et al., 2024; Achmad et al., 2024; Aslam et al., 2026; Choudhary et al., 2024; Hinge et al., 2023; Nagaraja et al., 2021; Ribeiro et al., 2022; Shoman et al., 2026; Varshney et al., 2026; Wang et al., 2024). A hydrogel can be justified when its functions address one or more of these constraints. Mucoadhesion and *in situ* gelation can mitigate rapid clearance; a retaining matrix can localize a carrier; sustained release can prolong local availability; and a carrier-in-gel strategy can combine payload solubilization or protection with prolonged residence at the nasal interface (Abou-Taleb et al., 2024; Cunha et al., 2022; Huang et al., 2024; Ribeiro et al., 2022). Added dosage-form complexity without a demonstrated functional contribution is more difficult to justify, irrespective of material novelty.

This rationale is evident for symptomatic AD drugs. Rivastigmine studies repeatedly address low oral bioavailability, first-pass metabolism, short half-life, gastrointestinal effects, limited brain concentrations, or inadequate nasal retention (Abouhussein et al., 2018; Achmad et al., 2024; Aslam et al., 2026; Hinge et al., 2023; Wavikar et al., 2017; Wavikar and Vavia, 2015). Donepezil systems similarly seek to improve brain exposure or reduce limitations associated with hepatic metabolism, plasma fluctuations, gastrointestinal intolerance, peripheral toxicity, and adherence in older patients (Espinoza et al., 2022; Gangopadhyay et al., 2023; Gu et al., 2020; Rajput and Butani, 2022; Sonawane and Pokharkar, 2024a; Tuna et al., 2025; Wang et al., 2024; Zoupanou et al., 2025). Tacrine, galantamine, huperzine A, and memantine extend the same general route-optimization logic (Chen et al., 2019; Lotfi et al., 2025; Mohapatra et al., 2021; Qian et al., 2014; Shamarekh et al., 2020; Wang et al., 2011). In these cases, improved delivery may increase exposure or tolerability, but it should not itself be described as disease modification; any disease-relevant effect requires independent demonstration.

The rationale is broader and more heterogeneous for natural products, repurposed small molecules, and multicomponent preparations. Examples include quercetin, curcumin, lutein, sildenafil, felodipine, magnolol, scutellarin, naringin, vinpocetine, flurbiprofen, telmisartan, and a standardized polyherbal preparation (Aslam et al., 2026; Chin et al., 2021; Choudhary et al., 2024; Diddi et al., 2024; Hard et al., 2023; Jog et al., 2025; Ma et al., 2026; Mankar et al., 2026; Nagaraja et al., 2021; Ribeiro et al., 2022; Shoman et al., 2026; Varshney et al., 2026). Across these programs, the delivery rationale varies and may include poor solubility, instability, first-pass loss, short half-life, limited brain penetration, mucociliary clearance, or the need to sustain local availability; these limitations should therefore be attributed to the individual payload rather than generalized across the class. Related intranasal studies of azilsartan and ginseng further illustrate the broader attempt to combine brain-directed administration with neuroprotective or cognition-relevant pharmacology, although their evidentiary weight for hydrogel-specific effects depends on whether the gel contribution was directly characterized (Karmakar et al., 2026; Yadav et al., 2026). A hydrogel can therefore serve as an enabling technology for an otherwise difficult payload, but formulation rescue does not validate the therapeutic premise of the payload itself. This creates a distinct evidentiary burden: improved delivery must be distinguished from evidence that the delivered molecule produces an AD-relevant effect.

Biological and advanced payloads demonstrate a different value proposition. Insulin-containing systems emphasize controlled nasal delivery, distribution, biological activity, stability, and tolerability (Camlik et al., 2024; Picone et al., 2018). Extracellular-vesicle formulations use hydrogels to reduce rapid clearance and regulate release (Huang et al., 2024), whereas neprilysin-containing collagen hydrogel uses the matrix as a vehicle for an amyloid-degrading enzyme (Humpel, 2021). D-penicillamine hydrogel addresses delivery of a water-soluble metal chelator (Zhong et al., 2021), and black phosphorus–methylene blue hydrogel integrates controlled delivery with antioxidant, mitochondrial, inflammatory, and tau-directed functions (Liu et al., 2024). In such systems, payload protection and controlled local availability may be important alongside permeation enhancement. These systems integrate multiple functions, but each additional biological or responsive component may also increase the need for analytical control, reproducibility, safety characterization, and manufacturing consistency.

A useful cross-platform principle follows: the hydrogel should be justified by a defined failure mode of the payload or route. Established symptomatic drugs provide a relatively straightforward demonstration of this principle because their pharmacology is already clinically recognized and their delivery limitations are relatively well defined. Experimental payloads may offer greater mechanistic ambition, but they can combine uncertainty about the drug with uncertainty about the delivery system. Translational interpretation should therefore distinguish two questions that are often merged: “Does the hydrogel improve delivery?” and “Is the payload therapeutically valuable when delivered?”

## 4 Formulation architecture and development logic

### 4.1 From matrix-led gels to carrier-in-gel systems

The reviewed systems can be organized into three architectural levels. Matrix-led systems place the active agent directly within a responsive or mucoadhesive gel. Carrier-in-gel systems first engineer a nanoparticle, vesicle, lipid carrier, nanocrystal, or other carrier and then incorporate it into a hydrogel depot. Advanced systems add charge engineering, surface modification, covalent conjugation, chemical crosslinking, or biologically responsive release (Abouhussein et al., 2018; Aslam et al., 2026; Cunha et al., 2022; Hinge et al., 2023; Huang et al., 2024; Liu et al., 2024; Mazahir et al., 2025; Picone et al., 2016; Shoman et al., 2026; Wavikar and Vavia, 2015). The first two levels recur broadly across the supplied corpus; the third comprises heterogeneous, platform-specific demonstrations rather than a mature common strategy.

Poloxamer 407 (P407) is a recurrent thermogelling matrix and is often combined with Poloxamer 188 (P188), chitosan, Carbopol, hydroxypropyl methylcellulose (HPMC), gellan gum, sodium alginate, or other mucoadhesive polymers (Abouhussein et al., 2018; Adnet et al., 2020; Botan et al., 2024; Chen et al., 2019; Gu et al., 2020; Hard et al., 2025; Mathure et al., 2025; Tuna et al., 2025; Wavikar and Vavia, 2015; Wu et al., 2023). This recurrence suggests broad formulation familiarity and adaptability, but does not indicate that P407 is universally optimal. The design space nevertheless varies widely. A rivastigmine factorial study selected P407 with 1% Carbopol 934 after comparing poloxamer composition, mucoadhesive polymer type, and polymer concentration (Abouhussein et al., 2018). Other direct matrices include HPMC/P407 for meloxicam (Botan et al., 2024), P407/P188 systems for donepezil and vinpocetine (Gu et al., 2020; Hard et al., 2025), a dual temperature- and pH-responsive huperzine A microemulsion gel (Chen et al., 2019), a chitosan-based telmisartan nanoemulgel (Chin et al., 2021), an ion-triggered harmine nanocrystal gel (Huang et al., 2021), and chemically crosslinked berberine and black phosphorus–methylene blue systems (Liu et al., 2024; Mazahir et al., 2025). Hydrogel nanoparticles based on trimethyl chitosan and sodium alginate provide a distinct particulate architecture for progesterone delivery (Cardia et al., 2019); because the gel network is confined to the nanoparticle rather than a macroscopic nasal depot, such systems should be distinguished from conventional *in-situ* gels when making cross-platform comparisons. The major pattern is therefore one of functional pluralism: thermoresponsive systems recur across many studies, whereas ion-triggered, particulate-hydrogel, and covalently structured matrices address distinct formulation needs.

Carrier-in-gel systems divide functional tasks between components. Chitosan nanoparticles have been used for dihydroquercetin, quercetin–polyethylene glycol (PEG)/rivastigmine, vinpocetine, and memantine; thiolated chitosan has been used for galantamine; and polyvinyl alcohol (PVA)/chitosan nanofibers have been transformed into a rivastigmine hydrogel (Abou-Taleb et al., 2024; Aslam et al., 2026; Hard et al., 2023; Lotfi et al., 2025; Mohapatra et al., 2021; Yadav et al., 2024). Poly(lactic-co-glycolic acid) (PLGA)-based systems similarly combine particle engineering with a final P407 or Carbopol matrix (Salatin et al., 2017; Shamarekh et al., 2020). The implication is important: terms such as “chitosan hydrogel” or “PLGA hydrogel” can conceal materially different architectures. Polymer identity alone is therefore a poor basis for cross-study ranking; carrier preparation method, surface properties, matrix composition, final rheology, and interface performance must be considered together.

Lipid and vesicular systems introduce still more variables. Citicoline transbilosomes, conventional and modified liposomes, donepezil ethosomes, sildenafil novasomes, naringenin cubosomes, solid lipid nanoparticles, lipid microspheres, and nanostructured lipid carriers (NLCs) have all been embedded in gels (AbouElhassan et al., 2022; Adnet et al., 2020; Bahndare et al., 2025; Choudhary et al., 2024; Cunha et al., 2022; Gangopadhyay et al., 2023; Raut et al., 2025; Séguy et al., 2026; Shoman et al., 2026; Tuna et al., 2025; Wavikar et al., 2017). Several studies use design-of-experiments methods to evaluate lipid composition, surfactant concentration, processing conditions, and related variables (AbouElhassan et al., 2022; Bahndare et al., 2025; Choudhary et al., 2024; Shoman et al., 2026; Sonawane and Pokharkar, 2024a). NLCs containing rivastigmine, donepezil, quercetin, atomoxetine, empagliflozin, nifedipine, resveratrol, and ginseng illustrate the flexibility of the carrier-in-gel approach (Cunha et al., 2022; Al Harthi et al., 2019; Dighe et al., 2024; Mohanty et al., 2023; Puranik et al., 2026; Rajput et al., 2018; Sonawane and Pokharkar, 2024a; Sonawane and Pokharkar, 2024b; Wavikar et al., 2017; Yadav et al., 2026). The tegaserod program is also informative because both nanoemulsion and liposome candidates were considered before gel-embedded liposomes were selected for intranasal use on the basis of the reported rheological and handling profile (Séguy et al., 2026). The reviewed studies do not establish that increasing carrier complexity consistently improves overall final-product performance. Cationic versus anionic rivastigmine lipid–polymer hybrids and NLCs versus nanoemulsion systems provide useful formulation-specific contrasts, but do not establish a general hierarchy (Cunha et al., 2022; Hinge et al., 2023).

### 4.2 QbD terminology and the need to connect inputs to performance

The source corpus repeatedly uses factorial, Box–Behnken, central composite, response-surface, and related optimization designs (AbouElhassan et al., 2022; Abouhussein et al., 2018; Chen et al., 2019; Cunha et al., 2020; Karmakar et al., 2025; Kurangi et al., 2026; Mohanty et al., 2023; Shoman et al., 2026; Yadav et al., 2024). This is a strength, but statistical optimization should not automatically be equated with a complete quality-by-design program. One study of rivastigmine-loaded NLCs explicitly framed nose-to-brain delivery through a QTPP and sequentially optimized formulation variables and production conditions, selecting high-pressure homogenization after evaluating carrier CQAs and storage behavior (Cunha et al., 2020). Elsewhere, optimization in the reviewed studies often focuses on a narrower set of responses, such as particle size, entrapment efficiency, gelation temperature, release, or permeation.

For this Review, a development-oriented framework should map variables prospectively. Polymer grade and concentration, lipid-to-surfactant ratio, charge-modifying materials, permeation enhancers, carrier composition, and excipient levels are potential CMAs. Mixing conditions, homogenization pressure, sonication, spray-drying conditions, crosslinking, and other manufacturing settings are potential CPPs. Particle size distribution, drug content, encapsulation, pH, osmolarity, and rheology can function as CQAs when linked to the QTPP. Sprayability, gelation time, retention, release, permeation, and regional deposition are treated here as performance attributes indicating whether controlled CQAs produce the intended nasal behavior. This distinction avoids a conceptual shortcut in which every measured variable is labeled a CQA and every favorable biological response is treated as evidence of pharmaceutical quality.

The architecture should be no more complex than necessary. Advanced systems demonstrate that hydrogels can accommodate extracellular vesicles, enzymes, inorganic nanomaterials, and multistage targeting concepts (Fei et al., 2025; Huang et al., 2024; Humpel, 2021; Liu et al., 2024). However, each additional carrier, ligand, responsive bond, biological component, or crosslinking step can introduce additional CMAs, CPPs, analytical requirements, potential instability, and scale-up challenges. The development question is therefore not whether complexity can be engineered, but whether it provides a reproducible improvement in deposition, retention, payload protection, brain exposure, safety, or therapeutic effect that a simpler system cannot provide.

## 5 Nasal-interface performance: trade-offs define formulation readiness

### 5.1 Gelation, viscosity, sprayability, and retention are coupled variables

The hydrogel matrix earns its place in the dosage form only if it improves performance at the nasal interface. Across the corpus, pH, osmolarity, gelation temperature and time, viscosity, rheology, mucoadhesion, retention, deposition, release, permeation, stability, and local compatibility are reported with markedly different levels of detail (Adnet et al., 2020; Aslam et al., 2026; Cunha et al., 2022; Gangopadhyay et al., 2023; Karmakar et al., 2025; Mathure et al., 2025; Perkusic et al., 2023; Rajput and Butani, 2022; Shoman et al., 2026; Varshney et al., 2026). Particle size and high entrapment efficiency may demonstrate successful carrier preparation, but they do not establish that the final system can be delivered reproducibly, reach the intended nasal region, remain there, release its payload appropriately, or avoid mucosal injury.

Gelation illustrates the need to interpret formulation attributes as an integrated system rather than individually. Many thermosensitive formulations report transition temperatures within the range of 28.5 °C–35.4 °C for nasal administration (Achmad et al., 2024; Chen et al., 2019; Gangopadhyay et al., 2023; Hard et al., 2023; Karmakar et al., 2025; Ma et al., 2026; Mankar et al., 2026; Perkusic et al., 2023; Qian et al., 2014; Wang et al., 2024). Gelation time, however, is reported much less consistently in the reviewed research; selected examples range from approximately 15 s–31.6 s (Hard et al., 2023; Hard et al., 2025; Mathure et al., 2025). Gelation temperature alone cannot establish effective retention. A formulation that gels too early may compromise handling or device delivery, whereas delayed gelation may permit drainage or clearance before an effective depot forms. Similarly, greater viscosity after administration may improve residence but can impede spray formation, spreading, and regional deposition. These are not independent targets to be maximized; rather, they require an operating window that permits administration first and retention second.

Rheological findings support this trade-off. Meloxicam HPMC/poloxamer formulations showed temperature-dependent changes from dilatant to pseudoplastic behavior with viscoelasticity (Botan et al., 2024), whereas tacrine gel changed from a Newtonian liquid below its transition temperature to a non-flowing pseudoplastic gel after gelation (Qian et al., 2014). Galantamine and berberine systems demonstrated gel-like viscoelastic behavior (Lotfi et al., 2025; Mazahir et al., 2025). Such findings provide information beyond a single static viscosity value because they describe how a formulation may behave during dispensing, deformation, and residence.

Sprayability is especially important because it connects rheology with device performance. A chitosan-based donepezil thermogelling spray demonstrated that a formulation can remain sprayable while subsequently gelling and achieving 71.8% olfactory-region deposition in a three-dimensional (3D)-printed nasal model (Perkusic et al., 2023). In the reviewed research, sprayability is not routinely assessed, and suitability for intranasal use does not by itself establish compatibility with a specific pump, nozzle, actuation force, plume geometry, droplet-size distribution, or delivered-dose reproducibility. Device performance should therefore be treated explicitly as part of the dosage form rather than as a downstream implementation detail. Together, these coupled formulation and device requirements define a nasal-interface operating window rather than a set of independently maximized attributes (Figure 1).

**FIGURE 1:**
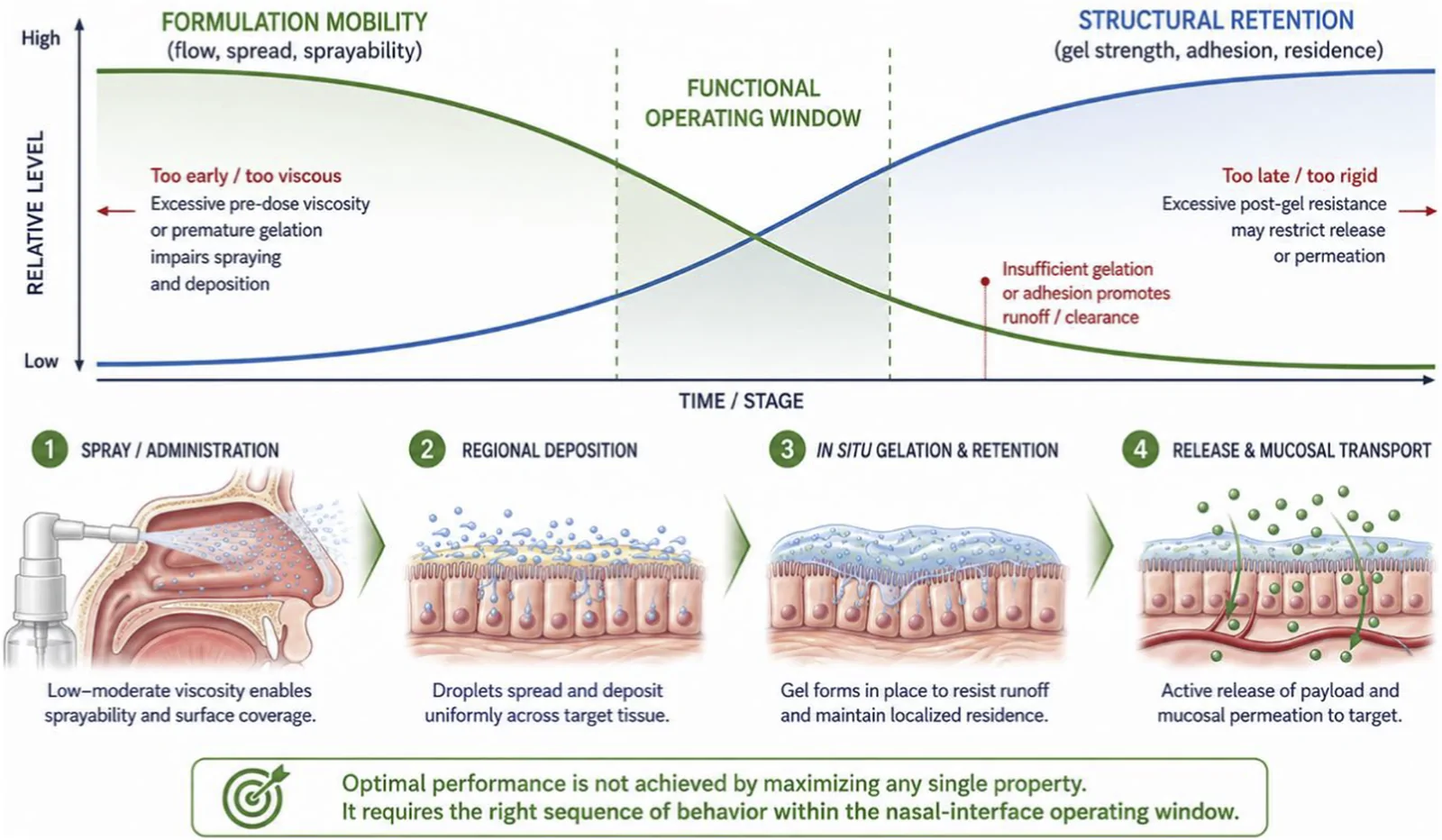
Nasal-interface operating window for intranasal hydrogel delivery. The formulation must remain sufficiently mobile for reproducible administration and anatomically relevant deposition before acquiring enough structure to resist clearance. The operating window therefore lies between two failure regimes: excessive pre-dose viscosity or premature gelation can impair spraying and regional deposition, whereas inadequate post-deposition gelation or adhesion promotes runoff and mucociliary loss. After retention is established, release must remain sufficient to support mucosal transport without requiring aggressive barrier disruption. The figure emphasizes temporal coordination—administration, deposition, gelation/retention, and release/permeation—rather than maximization of any single property.

### 5.2 Retention and deposition are more informative than mucoadhesion alone

Mucoadhesion is frequently invoked as a rationale for hydrogels, but its measurement is heterogeneous. Quantitative reports include mucoadhesion or adhesive-strength values for caffeic acid, donepezil, vinpocetine, and rivastigmine systems, while one rivastigmine solid lipid nanoparticle gel reported mucosal retention for 9–10 h (Das et al., 2026; Gangopadhyay et al., 2023; Hard et al., 2025; Sharma et al., 2026; Yadav et al., 2024). These data support the central hydrogel concept, yet the assays are not interchangeable and do not necessarily indicate where within the nasal cavity the formulation is retained.

Regional deposition is therefore a route-specific performance attribute. In a rivastigmine development program, incorporation into a gel improved olfactory deposition relative to the nanosystems alone, while a sprayable donepezil thermogel achieved 71.8% olfactory deposition in a three-dimensional (3D)-printed nasal cavity model and greater adhesion than solution (Cunha et al., 2022; Perkusic et al., 2023). These findings illustrate why anatomical deposition should complement conventional mucoadhesion testing. A formulation may be highly adhesive yet deposit predominantly in an anterior region that does not support the intended transport pathway.

The same logic applies to the apparent trade-off between residence and spreading. High viscosity and rapid gelation may reduce washout but may also restrict posterior distribution after administration. Lower viscosity may improve atomization or distribution but increase runoff and mucociliary loss. The desired balance is therefore route- and device-specific. The reviewed studies provide examples of each component of this relationship but do not establish that spray characteristics, deposition, retention, and pharmacokinetics were evaluated together. This incomplete linkage complicates the overall translational interpretation of otherwise sophisticated formulations.

### 5.3 Sustained release is useful only if sufficient drug becomes available for transport

Controlled or sustained release is repeatedly reported as a formulation advantage of hydrogel incorporation. Citicoline, insulin, galantamine, scutellarin, atomoxetine, metformin–curcumin, alpha-cyperone, donepezil, quercetin, rivastigmine, telmisartan, and ginseng systems all provide examples of controlled, sustained, or extended release (AbouElhassan et al., 2022; Aslam et al., 2026; Camlik et al., 2024; Chin et al., 2021; Lotfi et al., 2025; Mankar et al., 2026; Mohanty et al., 2023; Özyilmaz, 2026; Sonar et al., 2025; Tuna et al., 2025; Yadav et al., 2024; Yadav et al., 2026). Yet “slower” is not inherently “better.” If a matrix suppresses release too strongly during the period of nasal residence, only a limited fraction of the dose may become available for epithelial transport before the formulation is cleared or swallowed. Conversely, rapid release may compromise the residence advantage for which the hydrogel was introduced. The telmisartan nanoemulgel illustrates this balance: drug release was slower than from the corresponding nanoemulsion and varied with chitosan molecular weight and concentration, reinforcing that polymer selection can materially alter the release window rather than merely increase viscosity (Chin et al., 2021).

Particularly informative studies pair release measurements with *ex vivo* permeation or retention assessments. Comparator-based experiments with rivastigmine, surface-modified berberine, donepezil liposomes, caffeic acid, sildenafil novasomes, and curcumin-loaded mesoporous silica demonstrate that formulation changes can materially alter mucosal transport (Abouhussein et al., 2018; Bahndare et al., 2025; Rajput and Butani, 2022; Ribeiro et al., 2022; Sharma et al., 2026; Shoman et al., 2026). Such matched comparisons provide more direct evidence of formulation performance than unanchored release percentages because they can test the incremental contribution of carrier or hydrogel design.

Permeation enhancement may create a safety trade-off. Materials or formulations that increase epithelial transport may alter membrane integrity or tight-junction behavior. The osthole/borneol system, for example, was associated with changes in tight-junction-related proteins as part of its proposed mechanism for enhanced transport (Wu et al., 2023). Such effects may be mechanistically useful but increase the importance of repeated-dose epithelial and nasal safety testing. Permeation should therefore not be optimized independently of tolerability.

### 5.4 Stability and local compatibility remain underdeveloped product requirements

Selected systems provide stability evidence, including greater stability of donepezil liposomal hydrogel at 4 °C than at 20 °C, 90-day stability in a donepezil albumin nanoparticle development program, and 6-month maintenance of carrier attributes in insulin-loaded carbon quantum dot systems (Al Harthi et al., 2019; Camlik et al., 2024; Mathure et al., 2025). Structural and compatibility methods such as Fourier-transform infrared spectroscopy (FTIR), differential scanning calorimetry (DSC), X-ray diffraction (XRD), nuclear magnetic resonance (NMR), microscopy, and rheology recur across studies (Aslam et al., 2026; Hard et al., 2023; Lotfi et al., 2025; Mathure et al., 2025; Zoupanou et al., 2025). These analyses are valuable but should not be conflated with stability of the final drug product under clinically relevant storage conditions. Carrier stability alone does not establish preservation of gelation behavior, delivered dose, microbiological quality, or device performance.

Selected local tolerability findings are encouraging but fragmented. The hen’s egg test on the chorioallantoic membrane (HET-CAM), hemolysis, nasal ciliotoxicity, human nasal cell-tolerance studies, *ex vivo* biocompatibility, slug mucosal irritation, nasal histology, and systemic biochemical or hematological assessments have been applied to selected formulations (Achmad et al., 2024; Espinoza et al., 2022; Jog et al., 2025; Lotfi et al., 2025; Mazahir et al., 2025; Perkusic et al., 2023; Picone et al., 2018; Rajput and Butani, 2022; Wavikar et al., 2017). The diversity of these assays does not provide a common benchmark. More importantly, short-duration compatibility does not address the clinically relevant question of chronic exposure. A credible product therefore requires a linked performance package comprising controllable material and process variables, reproducible device delivery, suitable deposition and retention, adequate release and permeation, stability of the final dosage form, and repeated-dose mucosal and nasal safety.

The coupled performance variables and their quantitative anchors are summarized in Table 1.

**TABLE 1:** Coupled nasal-interface performance attributes and quantitative trade-offs that should govern intranasal hydrogel optimization.

Coupled design attributes	Quantitative evidence in the reviewed studies	Why optimization of one attribute alone is inadequate	Development implication	Representative references
Sprayability ↔ viscosity ↔ gelation	A sprayable donepezil thermogel gelled at 34 °C and achieved 71.8% olfactory deposition. An atomoxetine formulation containing NLCs had a viscosity of 2532 ± 18 cP at 37 °C and gel formation at 28 °C–34 °C	Increasing viscosity or accelerating gel formation may improve post-dose retention but can compromise spray formation, spreading, or dose reproducibility if structure develops too early	Evaluate pre-actuation rheology, device performance, gelation kinetics, and regional deposition as an integrated product-performance system	(Mohanty et al., 2023; Perkusic et al., 2023)
Gelation temperature ↔ gelation time ↔ retention	Reported gelation times included approximately 15 s, 23 ± 5.10 s, and 31.6 ± 1.52 s; selected systems reported adhesion for 7.22 ± 0.57 h or mucosal retention for 9–10 h	Transition temperature alone does not establish whether a depot forms quickly enough to resist clearance or so rapidly that spreading is impaired	Report gelation temperature and time together with a direct retention endpoint under relevant nasal conditions	(Das et al., 2026; Hard et al., 2023; Hard et al., 2025; Mathure et al., 2025)
Mucoadhesion ↔ regional deposition	A vinpocetine gel reported a mucoadhesive strength of 6,622 ± 2.64 dyne/cm^2^; a rivastigmine nanofiber hydrogel reported 236.7 ± 8.83 g/cm^2^. Olfactory deposition reached 8.0% after gel incorporation in a rivastigmine system and 71.8% in a sprayable donepezil system	Adhesion assays use non-interchangeable methods and units and do not establish where the dose is deposited	Treat mucoadhesion as evidence supporting residence and measure anatomical deposition separately when preferential nose-to-brain delivery is proposed	(Cunha et al., 2022; Hard et al., 2025; Perkusic et al., 2023; Yadav et al., 2024)
Sustained release ↔ fraction available for permeation	At 3 h, insulin release was 70.75% from solution versus 37.51% from the carbon-quantum-dot *in situ* gel system; other gels provided controlled release over periods of 8–24 h	Excessively slow release may leave insufficient drug available for transport during nasal residence, whereas rapid release can sacrifice the residence advantage	Define release targets in relation to retention and permeation rather than maximizing release duration in isolation	(Camlik et al., 2024; Lotfi et al., 2025; Yadav et al., 2024)
Permeation enhancement ↔ epithelial tolerability	Caffeic acid gel increased the permeability coefficient approximately 15-fold versus free drug, and sildenafil gel increased permeation 2.31-fold versus suspension; a berberine hydrogel maintained >80% cell viability	Transport-enhancing mechanisms may alter epithelial or tight-junction behavior, so a permeation advantage without local safety evidence is incomplete	Pair permeation assessment with measures of epithelial integrity, ciliotoxicity, irritation, histology, and repeated-dose safety when barrier modulation is plausible	(Mazahir et al., 2025; Sharma et al., 2026; Shoman et al., 2026; Wu et al., 2023)
Carrier stability ↔ complete-product stability	Donepezil liposomal formulations were more stable at 4 °C than at 20 °C; a donepezil albumin nanoparticle program reported 90-day stability; insulin-loaded carbon-quantum-dot systems maintained reported carrier attributes over 6 months	Carrier stability does not establish preservation of gelation, rheology, delivered dose, microbial quality, spray performance, or device compatibility	Extend stability programs from carrier-centered measurements to the complete drug–gel–device product	(Al Harthi et al., 2019; Camlik et al., 2024; Mathure et al., 2025)

## 6 Brain targeting and pharmacokinetic evidence

Brain-targeting claims are strongest when they combine a relevant comparator with a direct central-exposure measure. Rivastigmine and donepezil provide some of the clearest examples because hydrogel-associated formulations were compared with intranasal solutions, intravenous solutions, oral products, or intragastric controls (Abouhussein et al., 2018; Al Harthi et al., 2019; Gu et al., 2020; Wavikar et al., 2017). These comparisons are more informative than absolute concentrations without matched controls because they test whether formulation design contributes value beyond route selection alone. The comparator logic required to distinguish route effects from carrier- and hydrogel-associated effects is summarized in Figure 2.

**FIGURE 2:**
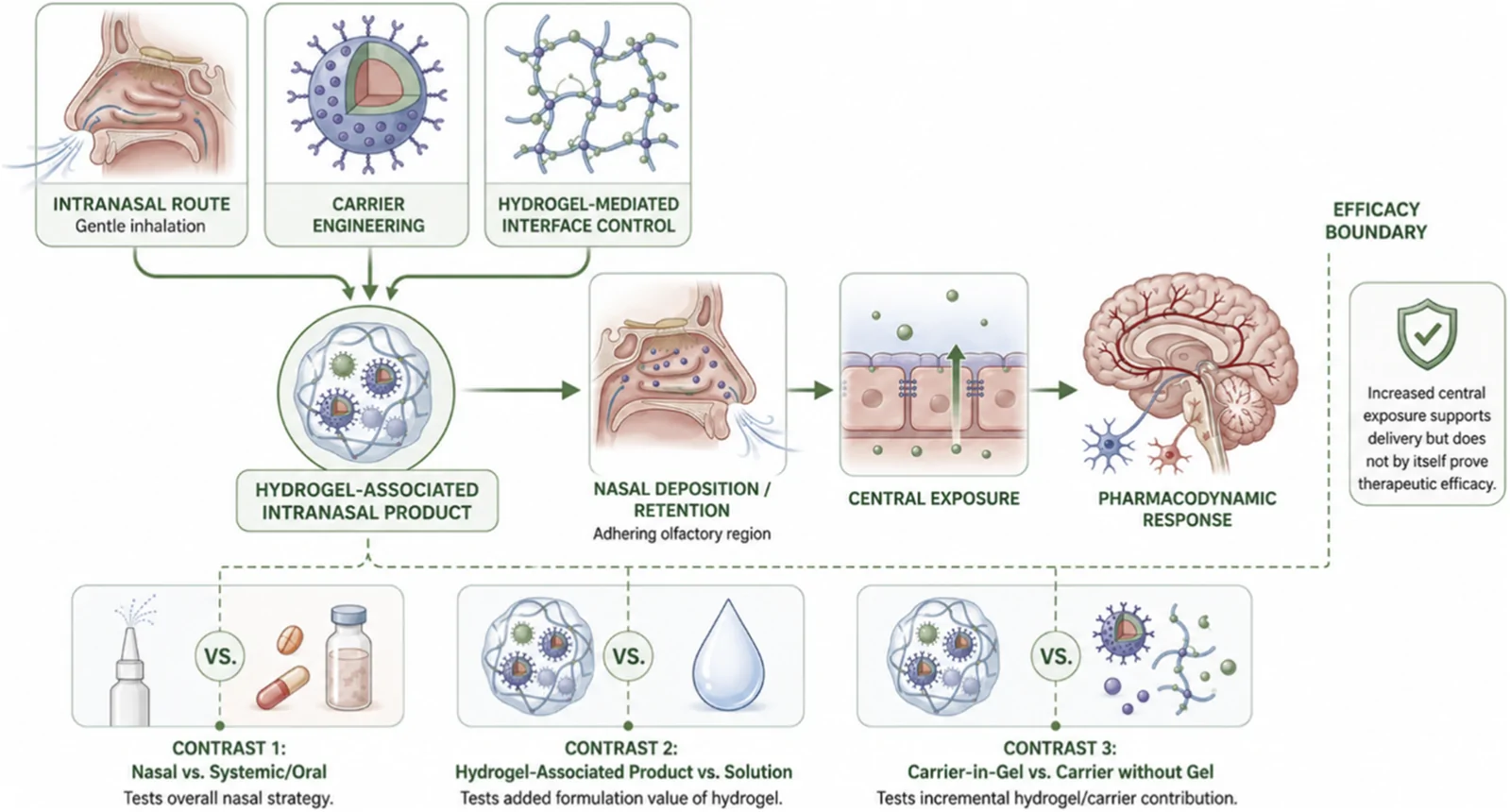
Comparator logic for attributing improved brain delivery to route, carrier, and hydrogel design. The final intranasal hydrogel-associated product reflects three potential contributors: the intranasal route, embedded carrier engineering, and hydrogel-mediated control at the nasal interface. Comparisons with oral or systemic formulations test the overall nasal strategy; comparisons with an intranasal solution test added formulation value; and comparisons between carrier-in-gel systems and the corresponding carrier without gel or a solution-based gel more specifically isolate the contribution of carrier or hydrogel incorporation. Evidence then progresses from nasal deposition/retention to quantitative central exposure and pharmacodynamic response. Improved brain exposure supports a delivery claim but does not independently establish therapeutic efficacy.

Within this framework, same-route comparisons in the same experimental design more directly test formulation effects beyond route selection, whereas cross-route comparisons reflect the combined effects of route and formulation differences. Accordingly, central-exposure results are interpreted below according to both endpoint directness and comparator structure rather than by absolute concentration alone.

The pattern extends beyond cholinesterase inhibitors but remains comparator-dependent. Flurbiprofen, vinpocetine, harmine, tacrine, protamine-coated tacrine, progesterone hydrogel nanoparticles, and ginseng-loaded NLC gel formulations reported higher central exposure or targeting relative to study-specific baselines or comparators (Cardia et al., 2019; Choudhary et al., 2024; Hard et al., 2023; Hard et al., 2025; Huang et al., 2021; Qian et al., 2014; Shamarekh et al., 2020; Yadav et al., 2026). These findings support formulation- or route-associated differences in brain delivery, but differences in species, dose, matrices, sampling times, tissues, units, and comparators preclude quantitative ranking across the reviewed platforms. Adjacent route-mechanistic studies reinforce the plausibility of direct intranasal transport without proving a hydrogel-specific effect: polymeric melatonin gels increased olfactory-bulb exposure relative to intravenous dosing (Babu et al., 2011), whereas thermoresponsive insulin nanoparticles showed prolonged nasal retention and a higher brain-to-serum distribution than insulin alone (Khan et al., 2026).

Derived targeting metrics require particular caution. The corpus reports drug-targeting efficiency (DTE), drug-targeting potential (DTP), drug-targeting index (DTI), and direct transport percentage across different included studies (Bahndare et al., 2025; Bhavna et al., 2014; Mathure et al., 2025; Puranik et al., 2026; Sonawane and Pokharkar, 2024a; Sonawane and Pokharkar, 2024b). The master evidence records do not preserve the original equations used by each publication; therefore, no retrospectively harmonized formula was imposed, and no cross-study recalculation was attempted. Reported values should be interpreted only within the calculation and comparator framework of the original study. This is especially important for reference (Mathure et al., 2025), in which the supplied record reports a DTE of 148% for both the nanoparticle gel and intranasal solution despite different DTI and DTP values. Likewise, the direct transport value reported in an adjacent non-hydrogel nanoparticle study should not be generalized to the hydrogel literature (Bhavna et al., 2014).

Imaging and deposition provide supportive rather than definitive evidence. Fluorescence, regional distribution, olfactory deposition, and brain localization strengthen biological plausibility (Chen et al., 2020; Cunha et al., 2022; Ma et al., 2026; Perkusic et al., 2023; Rajamma et al., 2022; Rajput and Butani, 2019), but they are not equivalent to brain AUC, C_max_, cerebrospinal fluid exposure, or route-matched biodistribution. Future studies should directly connect device-dependent nasal deposition with central pharmacokinetics and systemic exposure within the same integrated experimental program.

Selected matched comparisons that directly illustrate the study-specific incremental contribution of formulation design are summarized in Table 2.

**TABLE 2:** Quantitative comparator evidence for the incremental contribution of hydrogel-associated intranasal formulation.

Development question	Comparator that permits the contribution to be evaluated	Quantitative finding	What the comparison establishes	Representative references
Can carrier-in-gel design slow early payload release?	Insulin solution versus insulin-loaded carbon quantum dots incorporated into an *in situ* gel	At 3 h, insulin release was 70.75% from solution versus 37.51% from the gel-associated system	Supports more controlled early payload release; it does not establish brain targeting or therapeutic superiority	(Camlik et al., 2024)
Does adding gel improve anatomically relevant deposition beyond the nanosystem alone?	Rivastigmine formulation containing NLCs or nanoemulsion without gel versus the corresponding gel-associated system	Olfactory deposition increased from 4.0% to 8.0% after gel incorporation	Directly isolates an incremental contribution of gel incorporation to regional deposition	(Cunha et al., 2022)
Does carrier engineering within a gel improve mucosal transport beyond a simpler gel?	Surface-modified berberine liposomal gel versus plain berberine gel	*Ex vivo* permeation was 71.94% versus 46.64%	Shows an additional transport benefit from carrier engineering within a gel dosage form	(Bahndare et al., 2025)
Can carrier-in-gel outperform a solution-based gel containing the same drug?	Donepezil liposome-based *in situ* gel versus donepezil solution-based *in situ* gel	Permeation across sheep nasal mucosa was 80.11% ± 7.77% versus 13.12% ± 4.84%	Provides formulation-level evidence that the liposomal component materially alters nasal-interface performance even when both formulations are gels	(Rajput and Butani, 2022)
Does hydrogel dispersion improve transport beyond a preformed nanocarrier alone?	Curcumin-loaded mesoporous silica nanoparticles versus the same nanoparticles dispersed in hydrogel	Permeation increased from 12.46 ± 1.08 to 28.40 ± 1.88 μg/cm^2^	Demonstrates an incremental benefit attributable to the final hydrogel-associated dosage form rather than to nanoparticle preparation alone	(Ribeiro et al., 2022)
Can hydrogel-associated nasal dosing increase brain distribution relative to intranasal and systemic simple solutions?	Intranasal rivastigmine gel versus intranasal and intravenous rivastigmine solutions	Brain distribution was 0.54% injected dose/g versus 0.16% injected dose/g and 0.15% injected dose/g, respectively	Provides direct evidence that formulation design contributes beyond nasal administration alone	(Abouhussein et al., 2018)
Can higher central exposure accompany lower systemic peak exposure?	Intranasal flurbiprofen solid lipid nanoparticle (SLN) thermogel at 2 mg/kg versus oral marketed flurbiprofen at 10 mg/kg	Brain C_max_ was 490.3 versus 145.1 ng/mL; brain T_max_ was 0.5 versus 2 h; plasma C_max_ was 2.5 versus 3.4 μg/mL	Shows higher brain C_max_ with lower plasma C_max_, despite the differing routes and doses	(Choudhary et al., 2024)
Can one program show both nasal-interface and pharmacokinetic gains?	Lutein liposomal *in situ* gel versus solution-based gel for permeation and oral suspension for pharmacokinetics	Permeation was fivefold higher; study-reported DTE was 372.80%; dosing produced twofold higher C_max_ and 1.7-fold higher AUC than oral suspension	Shows concurrent interface and pharmacokinetic differences, while the use of different comparators prevents attribution of every effect specifically to the hydrogel alone	(Varshney et al., 2026)

## 7 Therapeutic evidence, mechanisms, and safety

Several hydrogel-associated systems improved memory, learning, escape latency, path length, exploratory behavior, object recognition, or spontaneous activity in AD or AD-like models (Abou-Taleb et al., 2024; Chen et al., 2020; Diddi et al., 2024; Hard et al., 2025; Karmakar et al., 2025; Kurangi et al., 2026; Mankar et al., 2026; Puranik et al., 2026; Shoman et al., 2026; Wang et al., 2011; Wavikar et al., 2017; Wu et al., 2023). More informative studies pair functional improvement with disease-relevant biological evidence. Dihydroquercetin nanoparticle gel improved behavioral outcomes while reducing amyloid formation, tau hyperphosphorylation, acetylcholinesterase activity, and oxidative stress, with accompanying histopathological improvement (Abou-Taleb et al., 2024). Citicoline thermogel reduced Aβ_1_–_42_ expression, inflammatory and oxidative signals, and tissue damage (AbouElhassan et al., 2022). Sildenafil, osthole/borneol, dauricine, taxifolin, berberine, a polyherbal *in-situ* gel, and huperzine A nasal gel show effects involving oxidative, inflammatory, mitochondrial, apoptotic, cholinergic, amyloid, tau, or APP-processing pathways (Diddi et al., 2024; Liu et al., 2024; Mazahir et al., 2025; Shoman et al., 2026; Wang et al., 2011; Wu et al., 2023; Xie R. Y. et al., 2026; Zhang et al., 2026). Additional intranasal studies of azilsartan and chitosan-coated ferulic-acid solid lipid nanoparticles report behavioral and biochemical improvement in AD models (Karmakar et al., 2026; Saini et al., 2021), but they should be interpreted as route- or carrier-level evidence unless the incremental contribution of a hydrogel is directly resolved. Likewise, gellan-derived midi-GAGR and mini-GAGR produced neurotrophic, antioxidant, amyloid/tau, and memory-related effects after intranasal dosing (Makani et al., 2016; Murphy et al., 2018); these studies are mechanistically relevant to intranasal brain delivery but are not evidence for a conventional hydrogel dosage form. Collectively, these results strengthen preclinical plausibility while preserving the distinction between hydrogel-specific, carrier-specific, and route-specific effects.

Mechanistic findings must not be overinterpreted. Amyloid- and tau-related effects, altered amyloid precursor protein (APP) processing, acetylcholinesterase inhibition, antioxidant responses, and mitochondrial signaling are biologically relevant, but pathway modulation is not synonymous with clinical disease modification. The neprilysin study is instructive: collagen-associated delivery supported plaque clearance, yet intranasal neprilysin alone was also associated with increased small amyloid deposits in selected cortical regions (Humpel, 2021). Such conflicting findings argue against mechanistic simplification and demonstrate why favorable biomarker changes must be interpreted alongside functional, tissue-level, and safety outcomes.

Several supplied studies remain delivery-dominant rather than efficacy-dominant in emphasis. Some studies emphasize retention, release, permeation, stability, biodistribution, or pharmacokinetic improvement without preserving defined cognitive or histopathological benefits (Al Harthi et al., 2019; Choudhary et al., 2024; Cunha et al., 2022; Gangopadhyay et al., 2023; Mathure et al., 2025; Raut et al., 2025; Tuna et al., 2025; Yadav et al., 2024; Zoupanou et al., 2025). These studies are valuable for pharmaceutical development, but they should be described as evidence of platform feasibility rather than therapeutic proof. Conversely, biologically promising formulations that lack robust characterization of deposition, rheology, stability, or repeated-dose tolerability require further development-focused product characterization.

Safety evidence is preliminary and assay-dependent. Selected systems show findings from HET-CAM, hemolysis, ciliotoxicity, cell viability, nasal histology, epithelial compatibility, or subacute toxicity assessments (Achmad et al., 2024; Jog et al., 2025; Mazahir et al., 2025; Perkusic et al., 2023; Picone et al., 2018; Rajput and Butani, 2022; Wavikar et al., 2017). A huperzine A nasal-gel study also reported no obvious toxicity during a 4-month intranasal treatment period in transgenic mice (Wang et al., 2011), providing useful longer-duration preclinical context without establishing chronic human nasal safety. However, chronic intranasal treatment for a neurodegenerative disease would require a substantially higher evidentiary standard. Repeated exposure should address epithelial integrity, mucociliary function, olfaction, local inflammation, immunogenicity where relevant, excipient burden, and the possibility that permeation-enhancing mechanisms alter mucosal barriers over time.

## 8 Human translation: from promising preclinical platform to product

The central translational weakness is the gap between preclinical administration and reproducible use in patients with AD. The supplied evidence is predominantly preclinical and includes rodents, *ex vivo* animal mucosa, cell systems, and formulation models. Human translation remains uncertain because patient anatomy, age, disease stage, comorbidities, adherence, and tolerability during chronic administration are not established by the reviewed research. These uncertainties should be treated as product-development variables rather than generic caveats.

Anatomical and administration differences matter because successful nose-to-brain delivery depends on where a formulation is deposited, how long it remains there, and how consistently that pattern can be reproduced. The studies include olfactory-deposition experiments (Cunha et al., 2022; Perkusic et al., 2023), but explicit studies of olfactory deposition are limited. Dose volume is also not preserved consistently enough to define a clinically credible administration window. Future programs therefore need to evaluate formulation volume together with rheology, plume or droplet behavior, device geometry, actuation, deposition, leakage or swallowing, and delivered-dose reproducibility. A hydrogel optimized in a vial is not a finished nasal product until its performance through the intended delivery device has been characterized.

The trade-offs discussed above become more consequential in human use. Higher viscosity may improve retention but impair atomization or dose reproducibility. Faster gelation may reduce clearance but increase the risk of premature gel formation within the device or before adequate spreading. Strong mucoadhesion may prolong contact yet does not guarantee deposition in an anatomically relevant region. Sustained release may be advantageous only if sufficient drug becomes available during residence, whereas aggressive permeation enhancement may challenge chronic mucosal safety. These relationships should be evaluated as an integrated design space rather than by optimizing one endpoint at a time.

Patient variability further limits simple extrapolation. Clinical translation should accommodate variation in age, disease stage, comorbidity, medication burden, ability to self-administer, and likely adherence. The reviewed studies do not consistently preserve evidence addressing age-appropriate, sex-inclusive, disease-relevant, and chronic-administration models before clinical translation. Reproducibility should therefore encompass not only batch-to-batch product quality but also dose-to-dose device performance and robustness to realistic variation in administration. Manufacturability, sterilization or microbial control, storage stability, sprayability, device compatibility, usability, nasal acceptability, and long-term safety require more comprehensive development-focused evaluation.

Accordingly, the translational endpoint should not be “brain targeting” in isolation. A credible development package would connect a justified QTPP with controlled CMAs and CPPs, final-product CQAs, device-compatible performance, anatomically relevant deposition, comparator-linked central pharmacokinetics, convergent pharmacodynamics, and repeated-dose safety. Until these elements are demonstrated together, the systems summarized here should be regarded as preclinical platforms rather than clinically validated AD therapies.

The resulting claim hierarchy—from formulation feasibility to translational readiness—is calibrated in Table 3.

**TABLE 3:** Quantitative evidence ladder for calibrating formulation, brain-targeting, therapeutic, and translational claims.

Evidence level	Representative quantitative anchor	Strongest defensible interpretation	Claim that remains unjustified at this level	Representative references
1. Integrated formulation and nasal-interface performance	A rivastigmine nanofiber-reinforced hydrogel showed gelation at 34.2 °C, pH 5.9 ± 0.12, spreadability of 19.51 ± 1.75 cm^2^, mucoadhesion of 236.7 ± 8.83 g/cm^2^, and controlled release over 8 h; the optimized nanofiber batch showed cumulative release of 90.17% ± 2.64%	Supports pharmaceutical plausibility because matrix behavior, mucosal interaction, and release are characterized together	Does not establish preferential brain delivery or therapeutic benefit	(Yadav et al., 2024)
2. Anatomical route-access evidence	A sprayable donepezil thermogel achieved 71.8% olfactory deposition and a 20-fold increase in adhesion relative to donepezil solution	Supports anatomically relevant olfactory deposition together with independent evidence of formulation adhesion	Does not establish preferential central exposure because deposition is upstream of brain pharmacokinetics	(Perkusic et al., 2023)
3. Comparator-resolved central exposure	An intranasal rivastigmine formulation containing cationic NLCs produced a brain concentration of 736.42 ± 34.54 ng/mL, reported as a 5.3-fold enhancement versus intranasal solution	Supports formulation-dependent improvement in brain exposure with a route-relevant comparator	Alone, does not establish cognitive efficacy, disease modification, or human benefit	(Wavikar et al., 2017)
4. Preferential-delivery metrics within a defined study	Donepezil gel containing NLCs: CNS C_max_ 193.41 ± 26.4 ng/mL, T_max_ 2 h, DTE 213.123%, DTP 66.27%. Quercetin gel containing NLCs: C_max_ 183.41 ± 11.76 ng/mL, T_max_ 2 h, DTE 117.47%, DTP 88.9%	Supports preferential-delivery interpretation within each study’s own calculation and comparator framework	Does not justify ranking the systems or comparing DTE/DTP values across studies unless the calculations and comparators are harmonized	(Sonawane and Pokharkar, 2024a; Sonawane and Pokharkar, 2024b)
5. Convergent preclinical therapeutic evidence	Scutellarin nasal gel improved spatial learning by 18.4% and reduced acetylcholinesterase activity by 25.9% versus pure scutellarin	Behavioral improvement supported by a disease-relevant biochemical endpoint strengthens preclinical therapeutic credibility beyond delivery evidence alone	Does not establish clinical efficacy or prove disease modification in human AD.	(Mankar et al., 2026)
6. Translationally informative but still preclinical evidence	Across representative programs, findings separately include 71.8% model-based olfactory deposition with acceptable irritation, no apparent nasal mucosal alteration or immunogenic response, and increased brain exposure without evident nasal epithelial damage in subacute testing	Supports feasibility across deposition, tolerability, and central-exposure domains without establishing readiness for clinical use	Human deposition reproducibility, chronic nasal and olfactory safety, manufacturability, patient variability, dose delivery, and therapeutic efficacy are not established by the reviewed studies	(Perkusic et al., 2023; Picone et al., 2018; Wavikar et al., 2017)

## 9 Limitations and future priorities

This evidence base is limited less by a shortage of formulations than by incomplete integration across the development pathway. The reviewed studies preserve particle size and entrapment more consistently than osmolarity, rheology, gelation time, deposition, long-term stability, device performance, and repeated-dose tolerability (Adnet et al., 2020; Botan et al., 2024; Camlik et al., 2024; Cunha et al., 2022; Hard et al., 2023; Kurangi et al., 2026; Mathure et al., 2025; Perkusic et al., 2023; Wavikar et al., 2017; Yadav et al., 2024). Comparator selection and pharmacokinetic endpoints vary across studies, and inferential statistics are unevenly represented. These limitations do not support ranking polymers, carriers, surface modifications, or responsive mechanisms.

Future work should therefore prioritize standardized evidence packages rather than additional architectural complexity. More informative studies could compare simplified and complex systems under matched conditions, define formulation and process variables prospectively, quantify regional deposition and retention, use comparator-resolved brain and systemic pharmacokinetics, and link these findings to disease-relevant pharmacodynamics and chronic nasal safety. Adjacent cognitive-disorder models and route-mechanistic intranasal systems can inform delivery principles but should not be used as direct evidence of hydrogel-specific AD efficacy when the final dosage form falls outside the operational hydrogel definition (Babu et al., 2011; Dwivedi et al., 2013; Elhelw et al., 2026; Fei et al., 2025; Khan et al., 2026; Li et al., 2024; Makani et al., 2016; Murphy et al., 2018; Parmar et al., 2026; Ruan et al., 2024; Saini et al., 2021; Xie Z. et al., 2026; Zhuang et al., 2024).

## 10 Conclusion

The principal challenge for intranasal hydrogel development in AD is no longer demonstrating that increasingly sophisticated formulations can be engineered, but establishing that each element of complexity produces a reproducible and clinically relevant improvement in delivery. A hydrogel is most defensible when it addresses a defined failure of the payload or route and when that contribution can be traced from administration and regional deposition through retention, release, permeation, and central exposure. The current literature provides important examples of such gains, but evidence remains fragmented across formulation characterization, pharmacokinetics, pharmacodynamics, device performance, stability, and safety. This fragmentation prevents meaningful ranking of polymers or carrier architectures and limits interpretation of targeting metrics, particularly when comparator design, experimental conditions, and calculation methods differ among studies. Translational progress will therefore depend on integrated development programs built around a justified QTPP, controlled CMAs and CPPs, relevant final-product CQAs, device-compatible administration, anatomically resolved deposition, matched brain and systemic pharmacokinetics, convergent disease-relevant outcomes, complete-product stability, and repeated-dose nasal and olfactory safety. Human variability, usability, manufacturability, and dose-to-dose reproducibility must ultimately be addressed within the same development framework. The field is therefore best positioned to advance not by adding unvalidated formulation complexity, but by converting promising preclinical systems into reproducible drug–gel–device products with evidence that remains coherent from pharmaceutical design through human use.
